# Decompressive effects of draining tube on suppurative and sclerosing osteomyelitis in the jaw

**DOI:** 10.1186/s12891-021-04340-3

**Published:** 2021-05-22

**Authors:** Buyanbileg Sodnom-Ish, Mi Young Eo, Mi Hyun Seo, Jong Ho Lee, Soung Min Kim

**Affiliations:** Department of Oral and Maxillofacial Surgery, Dental Research Institute, School of Dentistry, Seoul National University, 101 Daehak-ro, Jongno-gu, Seoul, 03080 South Korea

**Keywords:** Decompression, Suppurative osteomyelitis, Sclerosing osteomyelitis, Easy digitalized panoramic analysis (EDPA), Region of interest (ROI)

## Abstract

**Background:**

Osteomyelitis (OM) in the jaw is an inflammatory disease of osseous tissue that begins in the medullary space and progressively expands to the cortical portion of the bone, the Haversian system, the periosteum and the overlying soft tissue. Despite advances in dental and medical care, OM persists and is of important concern in modern medicine. Active negative pressure is known to prevent post-operative hematoma; decrease the number of bacterial pathogens, accumulation of toxins, and necrotic tissue; and promote osteogenesis and angiogenesis with the use of a draining tube such as the Jackson-Pratt (JP) or Hemovac. The purpose of this study was to assess the effectiveness of decompression for the treatment of OM in the jaw.

**Methods:**

This retrospective study included a total of 130 patients, 55 patients with sclerosing OM and 75 patients with suppurative OM were included. The radiographic bone densities expressed as a grayscale values (GSVs), were measured using an easy digitalized panoramic analysis (EDPA) method, processed on the conditional inference tree, generated by the R program® 3.2.3 with a probability of 96.8%. Rectangle annotation analysis of INFINITT PACS® (INFINITT Healthcare, Seoul, Korea) of 50 mm^2^ was determined as the region of interest (ROI). Student’s t-test and ANOVA were used to determine significance (*p* < 0.05).

**Results:**

Significant changes was observed between radiographic bone density in the sclerosing type with drain and without drain at the six-month and one-year follow-up (*p* < 0.05). Significant difference was demonstrated between the suppurative OM with drain and without drain groups at the one-year follow-up (*p* < 0.05).

**Conclusion:**

The OM groups with drain exhibited more enhanced bone density compared to the groups without drain at the six-month and one-year follow-ups. The drain insertion for decompression is effective for the management of sclerosing and suppurative OM. It is recommended to implement it for the management of OM.

## Background

Decompression and marsupialization are the major treatment options for odontogenic cysts. The efficacy of decompression has been widely demonstrated in odontogenic cystic conditions [1, 2]. Although the terms marsupialization and decompression have been used interchangeably, they are based on different concepts. Decompression effects can be classified as either passive (marsupialization and decompression) or active (negative pressure drainage) depending on the type of draining system used and the resulting induced negative pressure - low continuous, low intermittent, or high suction drainage [1]. Etymologically, the term marsupialization is derived from the Greek word *marsupial* meaning “pouch” [3]. Marsupialization is conversion of the cyst into a pouch, typically through suturing of the lining to the oral mucosa, so that the epithelial lining is left in situ [4]. Decompression on the other hand is the use of any draining tube (rubber tube, saliva ejector, catheter, anesthetic tube) to maintain patency between the inner contents of the cyst and the exterior environment [1, 5].

Decompression is based on the rationale that decreasing intraluminal pressure causes peripheral bone growth, changes the pathological environment, preserves anatomical structures, and prevents pathological fractures [6]. Recently, a two-way decompression device composed of two conduits, one of which is used for irrigation and the other for decompression has been introduced [2]. Castro-Nunez used an active negative pressure drainage apparatus connected to the internal compartment of the cyst to promote bone regeneration. The terms distraction sugosteogenesis and sugosteogenesis have been coined to define this negative pressure-induced osteogenesis [6].

Osteomyelitis (OM) in the jaw is an inflammatory disease of osseous tissue that begins in the medullary space and progressively expands to the cortical portion of the bone, the Haversian system, the periosteum and the overlying soft tissue. In the literature, the term OM has been used to describe multiple entities with different pathophysiological and clinical courses, resulting in confusion. The classification of OM varies among medical fields and is further obscured by radiographic, anatomic and etiological factors [7].

OM is typically caused by odontogenic infection or trauma. The odontogenic origin provides a direct pathway to the bone through pulpal infection or periodontal disease. This process begins with bacterial spread to the maxillary and mandibular bones, resulting in a bacteria-induced inflammatory disease [8]. Among the causative pathogens, *Staphylococcus aureus*, a facultative anaerobic gram-positive bacterium, is the most predominant in OM cases [9].

Among the various classification systems used for OM in the jaw, the Zurich classification is currently the most widely accepted. The Zurich Classification System differentiates the disease into three distinct entities: (1) acute OM, (2) secondary chronic OM, and (3) primary chronic (non-bacterial) OM [10]. The first two categories are characterized largely by suppurative nature, which is associated with sequestration and fistula formation [7]. Acute OM of the jaw develops over the course of several days to a few weeks and demonstrates a progressive onset of systemic symptoms, including fever, lymphadenopathy, leukocytosis, and edema formation in the affected region. Chronic OM is a relapsing and recurrent infection that progresses over months to years and is characterized by low-grade inflammation, presence of sequestration, new bone apposition and formation of fistulous tracts [11].

In the literature, two main variants of chronic OM are described. The suppurative variant has characteristics including presence of pus and or/fistulas and or/sequestrations that distinguish them from the non-suppurative variant, which is composed of chronic inflammatory processes of unknown etiology. The sclerosing variant is defined as an inflammatory bone condition of an uncertain origin even though it is generally believed that infections are of etiological significance. Radiographically, it is characterized by sequestra, sub-periosteal new bone formation, cortical defects, mixed sclerotic and osteolytic lesions are present and increased density of the medullary bone [12].

Despite advances in dental and medical care, OM persists and is of important concern in modern medicine. Consistent with the findings from previous authors, the prevalence of OM of the jaw seems to have increased recently [11, 13]. In addition, systemic diseases like diabetes, which predisposes individuals to OM partly due to decrease in vascularity of the bone, are becoming more common [13]. Due to the wide variety of classification systems, multiple surgical options, and unlimited options of antibiotic therapy, even the most experienced surgeons can have difficult deciding on optimal treatment for OM of the jaw. Although antibiotic therapy has helped augment such treatment, surgical treatment remains the main therapeutic interventions and includes debridement, decortication, and resection [13].

Following surgical debridement of necrotic bone tissue, placement of an irrigation and drainage system is important. Despite aggressive surgical therapies, the infection may remain as a deep seated and well-established condition harboring necrotic tissue that exhibits resistance to antibiotics and the immune system. Vacuum-assisted closure (VAC) or negative pressure wound therapy is often used in nearly all surgical fields for complex wounds to eliminate pooled blood, serum, and dead space except during head and neck surgeries. VAC is seldom used in this field due to the complexity of the anatomical structures and conjunction of head and neck wounds with heavy bacterial burdens such as orocutaneous fistulae [7]. With installation of saline solutions, this treatment method is even more effective in infected soft-tissue wounds through enhancement of wound healing and reduction of wound infections [8, 9]. Ceasar et al. reported use of a closed double-lumen suction irrigation system in chronic OM cases involving long bones. The suction and drainage aspects of this system allow for a “safety net” where the microbiology, volume, and appearance of the drainage fluid can be analyzed. Clear drainage indicates formation of healthy granulation tissue; if cloudy drainage persists, further surgical debridement must be undertaken, making way for proactive surgery. Other advantages included recovery of additional organisms from deep tissues [14]. A subjective method for evaluating the bone healing process in cystic lesions is associated with imprecise results and produces a significant bias that can be prevented by using the computerized analysis method [15]. Therefore, we implemented a picture archiving and communication system INFINITT PACS® (INFINITT Healthcare, Seoul, Korea) to measure radiographic bone density.

The purpose of this retrospective study was to quantitatively evaluate the effectiveness of decompression in treatment of chronic OM in the jaw using decompression with drain. We also discussed management updates about OM pathology and present management trends.

## Methods

### Patients and study design

From January 2009 through January 2020, 130 patients with OM who underwent treatment at Seoul National University Dental Hospital were retrospectively analyzed. OM of the jaw was classified as either suppurative or sclerosing type [16]. All panoramic radiographs were obtained with an Orthopantomograph OP100® (Instrumentarium Corp., Helsinki, Finland). The current study and its access to patient records were ethically approved by the Seoul National University Institutional Review Board (S-D20160039). All patients with OM in the jaw and odontogenic lesion were diagnosed by one oral and maxillofacial surgeon and included into the retrospective study with no age limit. Diagnoses for OM in the jaw were established based on patient medical history, clinical evaluation, radiological findings, and biopsy results.

Patients with OM were classified into four groups: sclerosing type with drain, sclerosing type without drain, suppurative type with drain, and suppurative type without drain.

#### Inclusion criterion

1. Patients with OM of the jaw who were treated at our institution between January 2009 and January 2020.
2. Patients diagnosed with sclerosing OM and suppurative OM in the jaw.
3. Patients with full clinical data, including the periodic panoramic radiograph and laboratory data.

#### Exclusion criteria

1. Patients with insufficient medical records for analysis such as panoramic radiographs, laboratory data. and who were lost during the follow-up period.
2. Patients diagnosed with acute OM and other types of OM, such as the osteoradionecrosis, Bisphosphonate-related osteonecrosis of the jaw (BRONJ), Medication-related osteonecrosis of the jaw.
3. Patients who underwent partial resection and reconstruction.

### Treatment protocol and radiographic assessment

Surgical therapy is one of the major pillars for treatment of acute and secondary chronic OM. Surgical debridement of infected tissue and removal of infectious foci were performed. Based on progression and pathology of the disease, we performed surgical therapy with minor surgical procedures such as removal of infected teeth, dental implants, fistulectomy, sequestrectomy, saucerization, and decortication. Following debridement of the infected tissue, we placed a silastic and penrose drain for passive drainage and a Hemovac or Jackson-Pratt drain for active negative pressure to drain post-operative exudate and promoted bone regeneration in the decompression groups of sclerosing type with drain and suppurative type with drain. The average amount of pressure measured in Jackson-Pratt drain was less than 100 mmHg, while the Hemovac drain generated 71 ± 4 mmHg. The criterion for selecting the type of drain was based on the extent of the surgery and degree of marrow bone exposure. For extensive surgeries resulting in large amount of defect, a suction drain is more appropriate choice for the excess of removal of post-operative exudate. In smaller defects, a general tube is usually implemented.

When placing the decompressive drains in surgical wound area, the drains in situ should be placed between the soft tissue and the bone wall, avoiding from placing it into the bone cavity. This will serve as an easy method for fixture using suturing to the soft tissues and easy removal. Passive drainage and active negative pressure drainage were applied 1–5 days post-operatively.

### Bone density measurement method

**Fig. 1:**
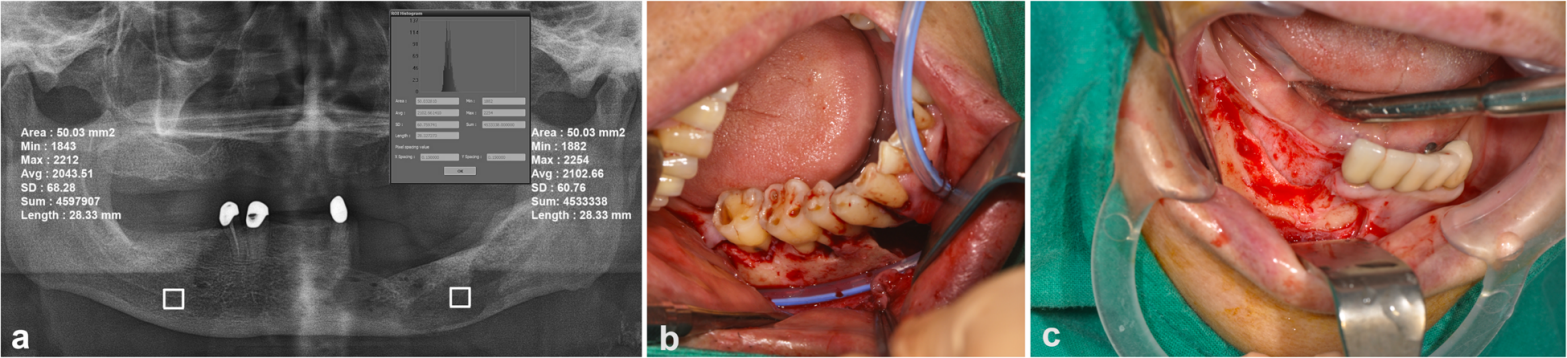
The radiographic bone density expressed as grayscale values (GSV) were measured by using the easy digitalized panoramic analysis method. Rectangle annotation analysis of INFINITT PACS® (INFINITT Healthcare, Seoul, Korea) was used for measuring the radiographic bone density GSV in suppurative OM as shown in patient No.7. A standard rectangular lesion area of approximately 50 mm^2^ was defined as the region of interest, designated at the center of the OM lesion. The bone density of the contralateral bone in the healthy region was also measured as a reference value to determine the absolute value (**a**). Intraoperative view of decompressive drain insertion intraorally following surgical management in suppurative type OM with drain (**b**). Intraoperative view of surgical debridement in sclerosing OM without drain (**c**)

The radiographic bone densities expressed as grayscale values (GSV) were measured using an easy digitalized panoramic analysis (EDPA) method. Park et al. reported a method for early diagnosis and classification for OM in the jaw, which was processed with a decision making tree generated on R program® version 3.2.3 for classifying the target group into subgroups based on the decision making tree with a probability of 96.8% [17]. We measured the GSV of the two groups on a fixed timeline. The measurements were collected immediately post-operatively and at three-month, six-month, and one-year follow-ups. We used INFINITT PACS® (INFINITT Healthcare, Seoul, Korea) to measure bone density, where the average and standard deviation of the pixel values can be measured within the ellipse, rectangle, or arbitrary shape of choice. Rectangle annotation analysis was used as a standard length and width for each measurement. A standard rectangular lesion area of approximately 50 mm^2^ was defined as the region of interest (ROI), designated at the center of the OM lesion (Fig. 1a). Areas of teeth, titanium plates, dental implants, and any other foreign bodies were excluded. The bone density of the contralateral bone in the healthy region was measured as a reference value to determine the absolute value since there was no standardization of absolute value due to an error converting three-dimensional (3D) data into two-dimensional (2D) data, which depends on the posture and timing of the radiographic image. The difference between the ROI and the healthy contralateral site was calculated immediately post-operatively, 3 months later, 6 months later, and 1 year later to evaluate bone healing in the affected areas. In the panoramic radiograph, minimum, maximum, and average variables are presented in the histogram with values ranging from − 240 to 2640, where low radiographic density is expressed as radiopacity, and high radiographic density is expressed as radiolucency. For measurement, we used the dependent average variable of radiographic bone density [17].

### Laboratory analysis

We retrospectively reviewed the medical records of all patients preoperatively and postoperatively. The hemoglobin (Hb), hematocrit (Hct), white blood cells (WBC), absolute neutrophil count (ANC), and erythrocyte sedimentation rate (ESR) from the standard routine laboratory analysis were obtained 1–7 days before surgery. The normal reference levels of Hb, Hct, WBC, ANC, and ESR were 12 ~ 16 g/dL, 39–52%, 4 ~ 10 × 10^3^/μl, 1800 ~ 7000/μl, and 0 ~ 20 mm/hr., respectively, at our institution. The postoperative laboratory results were compared with the preoperative results.

### Statistical considerations

All data were collected using Excel (Microsoft, USA), and statistical analysis was performed using SPSS 25.0® (SPSS Software Company, Chicago, IL, USA). Student’s t-test and ANOVA were used to determine significance, defined as 0.05 (*p* < 0.05), and in the comparisons between mean values.

## Results

### Patient and treatment data

**Fig. 2:**
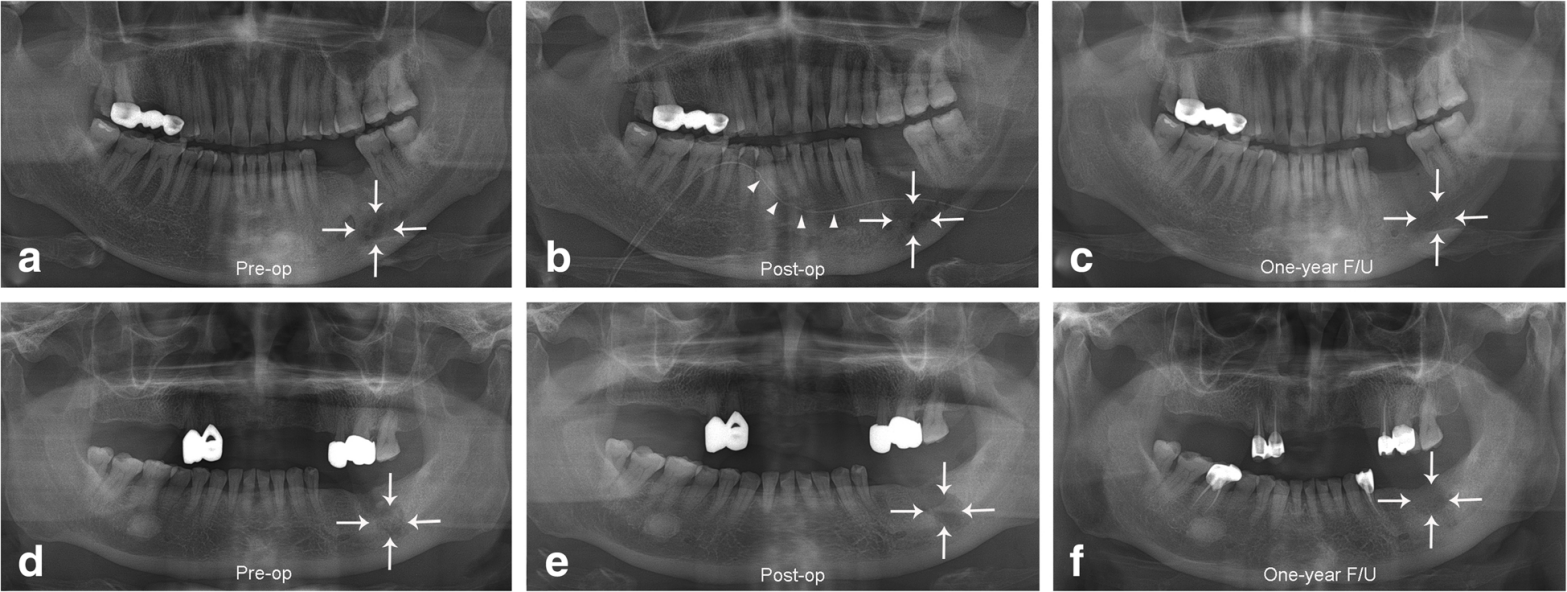
A panoramic radiograph showing the comparison of sclerosing type OM with and without drainage.. Pre-operative sclerosing OM on panoramic X-ray shows as a dense lesion with increased radiopacity compared to the surrounding healthy bone tissue (marked with arrows) in patient No. 22 (**a**). Immediate post-operative view following saucerization and drain insertion for decompression (marked with arrowheads). Panoramic X-ray view may show only the lingual wall of mandibular body with minimum soft tissue image. **b**. Following the decortication procedure, the sclerosing OM could be healed uneventfully with bone turnover, resulting in the decrease of bone density in the sclerotic area (the decrease of GSV) with minimal difference from the surrounding healthy bone tissue at one-year follow-up (**c**). Pre-operative panoramic radiograph showing sclerotic OM in the left posterior mandible region in patient No.67 (**d**). Immediate post-operative view without drain (**e**). One-year follow-up view (**f**)

**Fig. 3:**
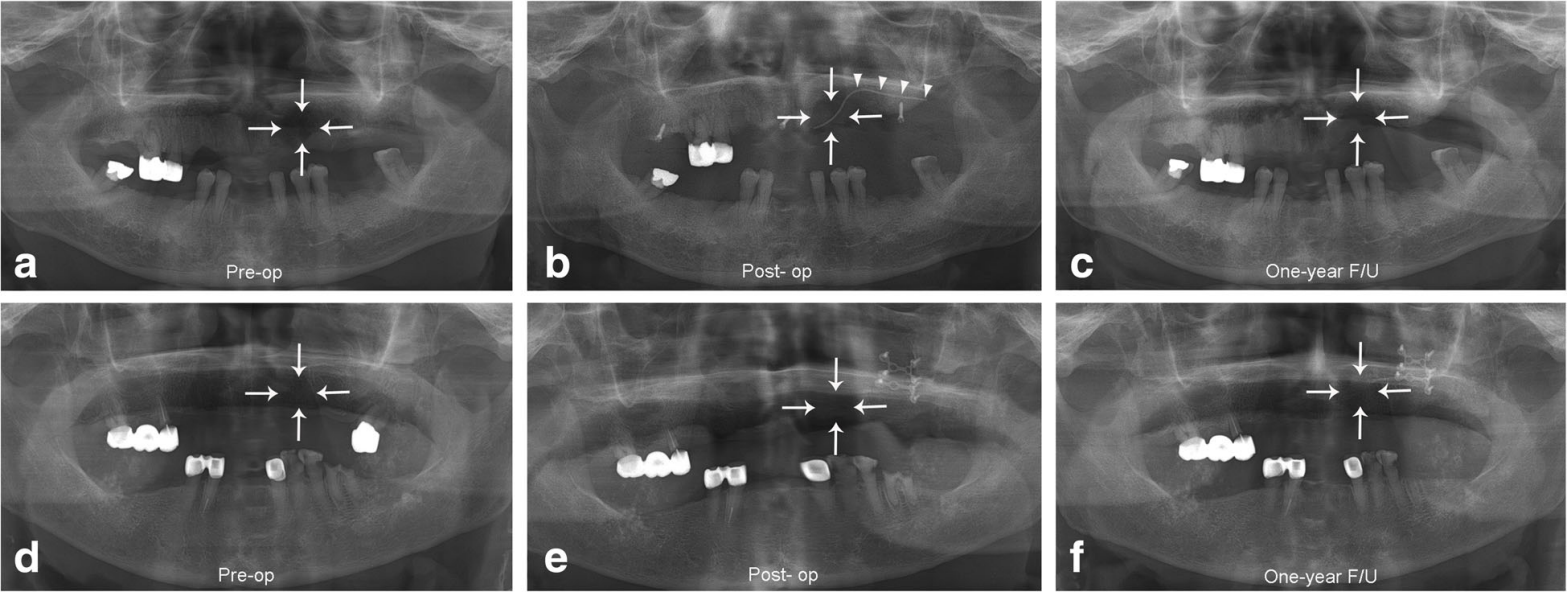
A panoramic radiograph comparing suppurative type OM with and without drainage. Pre-operative radiograph showing a suppurative OM lesion in the left posterior maxilla region (marked with arrows) in patient No. 30 (**a**). Immediate post-operative view following saucerization and drain insertion for decompression (marked with arrowheads) (**b**). A one-year follow-up radiograph showing bony healing (**c**). Pre-operative panoramic radiograph showing sclerotic OM in the left posterior maxilla region in patient No. 23 (**d**). Immediate post-operative view without drain (**e**). One-year follow-up view (**f**)

Based on the inclusion and exclusion criteria, a total of 43 patients were analyzed, in which the 23 patients were diagnosed with sclerosing OM, while 20 patients were diagnosed with suppurative OM. Among them, 12 cases were males and 31 were female patients, with a mean age of 64.4 ± 14.6 ranging from 12 to 87 years. Regarding site, the right posterior mandible was the most commonly affected (*n* = 19, 44.1%), followed by the left posterior mandible (*n* = 16, 37.2%), the anterior mandible (*n* = 3, 7%), the left posterior maxilla (*n* = 3, 7%), the anterior maxilla (*n* = 1, 2.3%), and the right posterior maxilla (*n* = 1, 2.3%). In our study, a total of 29 patients underwent decompression with drainage following the surgical procedures (Fig. 1b), and 14 patients without drainage were included (Fig. 1c). The patients were further categorized based on subtype of disease and use of a drain. In the sclerosing type OM group, 14 (32.6%) patients received decompression with drainage, while nine patients were treated without drainage (Fig. 2) (20.9%). In the suppurative type OM group, 15 patients received decompression treatment with drainage (34.9%), while five patients were treated without drainage (11.6%) (Fig. 3). The most frequently performed surgical treatment was saucerization (*n* = 34, 79%) followed distantly by sequestrectomy (*n* = 7, 16.2%) (Table 1).

**Table 1:** Clinicopathologic features of OM in this study

Variables	Sclerosing OM with drain (*n* = 14)	Sclerosing OM without drain (*n* = 9)	Suppurative OM with drain (*n* = 15)	Suppurative OM without drain (*n* = 5)
Sex				
Male	4	7	5	1
Female	10	2	10	4
Age	67.07 ± 10.83 (82–45)	61.89 ± 13.74 (83–37)	62.80 ± 17.29 (80–12)	66.60 ± 19.76 (87–40)
Signs and symptoms				
Swelling	6 (42.86%)	6 (66.67%)	11 (73.33%)	3 (60.00%)
Pain	9 (64.29%)	5 (55.55%)	7 (46.67%)	2 (40.00%)
Fistula	4 (28.57%)	–	5 (33.33%)	1 (20.00%)
Pus discharge	4 (28.57%)	3 (33.33%)	5 (33.33%)	1 (20.00%)
Tenderness	1 (7.14%)	–	2 (13.33%)	1 (20.00%)
Exposed necrotic bone	–	–	1 (6.67%)	–
Discomfort	1 (7.14%)	1 (11.11%)	–	1 (20.00%)
Etiology				
Odontogenic infection	11 (25.58%)	3 (6.97%)	8 (18.6%)	1 (2.32%)
Post-extraction complication	4 (9.30)	5 (11.63%)	5 (11.63%)	3 (6.97%)
Infected fracture	–	–	1 (2.32%)	–
Sinusitis	2 (4.5%)	–	–	–
Number of interventions	1.14 ± 0.36 times	1.22 ± 0.44 times	1.06 ± 0.01 times	1.40 ± 0.54 times
Systematic disease				
Hypertension	8 (57.14%)	4 (44.44%)	8 (53.33%)	1 (20.00%)
Diabetes mellitus	2 (14.29%)	1 (11.11%)	3 (20.00%)	2 (40.00%)
Bleeding tendency	2 (14.29%)	–	3 (20.00%)	–
Cardiovascular	2 (14.26%)	–	–	–
Osteoporosis	3 (21.43%)	2 (22.22%)	2 (13.33%)	1 (20.00%)
Rheumatoid arthritis	–	–	1 (6.67%)	–
Smoking	1 (7.14%)	–	1 (6.67%)	–
Time of drain in situ	2.14 ± 1.35 days	–	3.87 ± 1.53 days	

### Cases of radiographic bone density in OM of the jaw

We compared radiographic bone density changes between the groups immediately post-operatively and at the three-month, six-month, and one-year follow-ups. The immediate post-operative and three-month follow-up radiographic density results showed no significant differences between sclerosing type with and without drainage or between suppurative type with and without drainage.

**Fig. 4:**
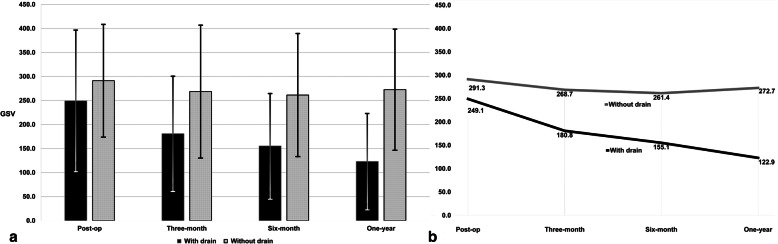
Chart presenting comparative radiographic bone density in sclerosing OM with drain and without drain. The typical findings of sclerotic OM in the jaw are mixed osteolytic and sclerotic lesions with periosteal new bone formation, therefore it is presented as radiopaque with lower photographic density than normal bone. Therefore, the difference between the ROI and the healthy contralateral side in the sclerosing OM is expressed as positive GSVs (**a**). Line graph presenting comparative bone density in sclerosing OM with and without drain. In the sclerosing OM with drain, bone density differences decreased with statistical significance from 249.1 ± 147.41 to 122.94 ± 100.08 GSV (*p* < 0.05) at the one-year follow-up. In the sclerosing OM without drain group, bone density changed from 291.28 ± 117.36 to 272.68 ± 126 GSV (*p* > 0.05) at the one-year follow-up. Statistically significant changes were observed between the sclerosing OM with drain and without drain groups (*p* < 0.05) (**b**)

In the sclerosing OM with drain, bone density decreased with statistical significance from 249.1 ± 147.41 to 122.94 ± 100.08 (*p* < 0.05) at the one-year follow-up. For evaluating the internal structure, the term ‘predominantly’ means more than half the lesion. For instance, where the internal structure of the lesion is predominantly more radiopaque than the surrounding bone, the lesion should be called sclerotic [12]. Therefore, bone healing in sclerotic OM indicates the normalization of the bone architecture, which results in radiopacity decrease of the lesion, making little difference from the surrounding healthy bone. In the sclerosing OM without drain, bone density differences changed from 291.28 ± 117.36 GSV to 272.68 ± 126 GSV (*p* > 0.05) at the one-year follow-up. In the OM cases, statistical significance was observed between radiographic bone density results in the sclerosing type with drain and without drain at the six-month follow-up (*p* < 0.05) and one-year follow-up (*p* < 0.05) (Table 2) (Fig. 4).

**Table 2:** Comparison of radiographic bone densities between sclerosing type with drain and sclerosing type without drain at different time periods

Sclerosing OM	With drain	Without drain	*P* - value
Immediately post-operative	249.1 ± 147.41	291.28 ± 117.36	0.478
Three-month follow-up	180.83 ± 119.99	268.67 ± 138.33	0.121
Six-month follow-up	155.13 ± 109.65	261.4 ± 128.25	0.046*
One-year follow-up	122.94 ± 100.08	272.68 ± 126	0.005*

*Statistical significance was performed with the t-test test (*p* < 0.05). ANOVA test to check if there are statistical significance between the groups at different time points. The absolute values for radiographic bone densities are presented as mean ± standard deviation grayscale value (GSV)

**Fig. 5:**
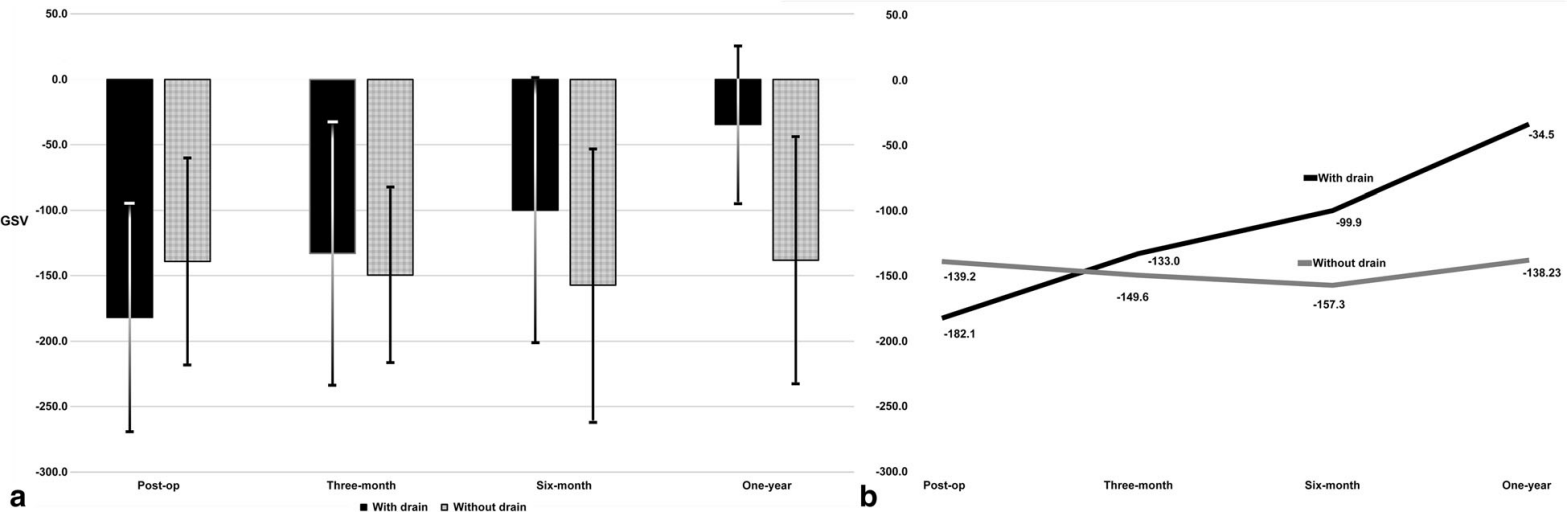
Chart presenting comparative radiographic bone density in suppurative OM with drain and without drain. Ill-defined radiolucent lesions of bony destruction and sequestrum formation are the typical findings of suppurative OM in the jaw. In this case, the GSV in the ROI is presented with higher photographic density values compared to normal bone. Therefore, the difference between the ROI and the contralateral side is expressed as negative GSVs (**a**). Line graph presenting comparative bone density in suppurative OM with and without drain. In the suppurative OM with drain, statistically significant change was observed between the post-operative and one-year follow-up radiographic bone density (*p* < 0.05). Statistically significant difference was observed between the suppurative OM with drain and without drain groups at the one-year follow-up, with a mean difference of − 149.73 ± 47.28 GSV (*p* < 0.05) (**b**)

In the suppurative type OM with drain group, statistically significant change was observed between the post-operative and one-year follow-up radiographic bone density (*p* < 0.05). Significant difference was observed between the suppurative OM with drain and without drain groups at the one-year follow-up, with a mean difference of − 149.73 ± 47.28 GSV (*p* < 0.05) (Table 3) (Fig. 5).

**Table 3:** Comparison of radiographic bone densities between suppurative type with drain and suppurative type without drain at different time periods

Suppurative OM	With drain	Without drain	*P* - value
Immediately post-operative	− 182.06 ± 86.38	− 139.2 ± 78.02	0.339
Three-months follow-up	− 133 ± 99.89	−149.61 ± 65.82	0.735
Six-months follow-up	−99.91 ± 100.22	− 157.5 ± 103.34	0.285
One-year follow-up	−34.51 ± 58.99	−138.21 ± 93.17	0.010*

* Statistical significance was performed with the t-test test (*p* < 0.05). ANOVA test to check if there are statistical significance between the groups at different time points. The absolute values for radiographic bone densities are presented as mean ± standard deviation grayscale value (GSV)

### Laboratory results in OM of the jaw

We analyzed pre- and post-operative blood tests including laboratory markers for systemic inflammation such as WBC, Hb, Hct, and ANC, as well as ESR. While there was no statistical difference in Hb, Hct, ANC or WBC pre- and post-operatively, the ESR exhibited statistically significant results over time in the OM with drain compared to the OM without drain (*p* < 0.05) (Table 4).

**Table 4:** Pre- and post-treatment blood test results of the OM cases

Group	Before treatment					After treatment				
	WBC	Hb	Hct	ANC	ESR	WBC	Hb	Hct	ANC	ESR
Sclerosing OM with drain	7.16 ± 2.63	11.46 ± 2.88	34.48 ± 8.35	4780.14 ± 2309.23	33.71 ± 28.29	6.24 ± 1.67	12.22 ± 2.18	36.55 ± 5.74	4144.1 ± 1777.74	14.1 ± 10.34
Sclerosing OM without drain	7.45 ± 2.2	12.26 ± 1.7	35.78 ± 8.13	4429 ± 2314.86	38.44 ± 23.80	6.56 ± 2.05	11.45 ± 2.09	35.03 ± 5.05	3555 ± 1872.67	26.66 ± 30.42
Suppurative OM with drain	7.85 ± 2.89	12.54 ± 2.34	32.94 ± 5.6	4964.73 ± 3134.15	48.8 ± 28.60	6.25 ± 1.83	12.21 ± 2.01	36.94 ± 5.20	4257.76 ± 2010.63	23.83 ± 19.02
Suppurative OM without drain	7.18 ± 2.9	12.41 ± 1.88	39.18 ± 5.63	6363 ± 2978.64	35.6 ± 19.90	6.89 ± 2.59	12.12 ± 2.15	37.33 ± 5.67	5217.66 ± 2411.95	32.66 ± 41.18

The results are presented as mean ± standard deviation

Abbreviations: *WBC* White blood cell count (× 1,000), *Hb* Hemoglobin, *Hct* Hematocrit, *ANC* Absolute neutrophil count, *ESR* Erythrocyte sedimentation rate

## Discussion

The currently acknowledged treatment for OM of the jaw is a combination of antibiotic therapy and surgical management with hyperbaric oxygen treatment. Between 500 and 700 species of bacteria have been identified in the human mouth [13]. Therefore, it should be no surprise that OM of the jaw is a polymicrobial infection. In a normal state, bone is highly resistant to infection. However, various in vitro experiments, animal models, and clinical studies have demonstrated that *S. aureus* is infection of osteoblasts and osteocytes with intracellular persistence within the bone. In this state, antibiotic delivery is limited due to the compromised local vascular system, and pathogenic bacteria may persist after debridement of necrotic bone tissue, which can be explained by the biofilm theory [8].

Recent trends in therapy of OM of the jaw are associated with early diagnosis and aggressive treatment options. This results in osseous defects and need for repair. In this case, more rapid wound healing and bone regeneration are required. The negative pressure decompressive drainage system is a cost-effective, time-efficient, and highly effective treatment option with low associated morbidity in cases of OM of the jaw. It is theorized that more enhanced bone regeneration and eradication of pathogenic bacteria can be achieved. With the use of this system, persisting pathogenic bacteria can be eliminated through negative pressure suction. With promotion of vasculogenesis, the antibiotic effects are enhanced. Passive and active negative pressure drainage systems have been routinely used for post-operative head and neck management. Although many surgeons use these drainage systems, there is a lack of clinical data on the bone regenerative aspects of decompression at OM sites.

The osteogenic properties of active negative pressure drainage have been widely reported in clinical cases of various odontogenic cysts, where they are used as a sole treatment in young developing patients where radical treatment should be avoided [3]. Despite the increasing number of reports on successful treatment of odontogenic cysts with decompression, there are no studies on the negative pressure drainage effects on OM of the jaw bone. The aim of our retrospective study was to evaluate the effectiveness of decompression using active negative pressure drainage in the bone healing process following surgical treatments in the OM of the jaw cases.

With regard to the demographic findings, OM lesions were most commonly found in female patients (*n* = 31, 72%), with the posterior mandible being the most prevalent site (*n* = 35, 81.39%). These results are consistent with previous studies regarding the body or angle of the mandible as the most prevalent site of infection [7]. Accordingly, OM in the maxilla is less frequent than that of the mandible, mainly due to the extensive maxillary blood supply [16].

In a recent study, quantitative assessment of panoramic radiograph using the PACS program was proven to be useful for early diagnosis and classification of OM in the jaw. According to this method, new patients showing symptoms can be considered suspicious of OM in the jaw with an average difference GSV greater than 54.49 and minimum value less than 31 or a difference average GSV between 12.81 and 54.49 and a difference minimum value of 39 [17]. Therefore, we used the EDPA method to evaluate the progress of bone healing by measuring radiographic bone density expressed as GSV. Panoramic radiographs are a major non-invasive method for detecting bone formation in a healing osseous defect. Bone healing is visualized by an increase in radiopacity, resulting in higher optical density [15]. However, in sclerosing type OM, the bone density is increased in lesions with high radiopacity [12]. In this case, the decrease of radiopacity compared to the initial density measurements is indications of normal bone healing in sclerotic type OM of the jaw. Significant results of reduction in density differences in the sclerosing OM cases site were observed at the six-month and one-year follow-ups. The sclerosing type with drain group exhibited a significant decrease in radiographic bone density differences compared to the sclerosing type without drain group at the six-month and one-year follow-ups (*p* < 0.05). Suppurative type OM of the jaw with drain demonstrated significant bone healing results of increased radiopacity at the affected lesion at one-year follow-up.

In this study it was found a significant difference in prognosis after 1–5 days of decompressive drainage at one-year follow-up. Although there was no significant difference in bone healing at three and six-months between the groups, the bone healing process was more gradual and consistent in the decompressive drainage groups. The surgical treatment included debridement of the necrotic and infected bone tissues from the marrow bone sparing the healthy periosteum and cortical bone walls as the sources of osteogenesis for bone regeneration. Research have shown that that suction drainage can expand the capillary diameter of wound, improving the microcirculation of wound, which brings more growth factors to the wounds and promotes the proliferation of granulation tissue [18]. Negative pressure suction drainage can promote the expression of multifarious repair signals and wound healing genes and increase the number of various growth factors and enzymes on the wound surface and surroundings, thereby promoting epithelial regeneration [19]. It should be noted that the complete bone healing of jawbone defects takes considerable amount of time. According to a clinical study by Hren et al., spontaneous bone healing of large bone defects occurred after 1 year, where 46% of bone gain was achieved after surgical treatment [20]. Thus, more bone healing occurred in cases where decompression drainage was applied compared to that of without drainage.

The sclerosing OM on panoramic X-ray shows as a dense lesion with increased radiopacity compared to the surrounding healthy bone tissue. The surgical treatment of sclerosing OM was usually done by decortication and removal of inflammatory debris and followed by drain application and periosteal soft tissue covering. Therefore, panoramic X-ray view may show only the lingual wall of mandibular body with minimum soft tissue image. Following the decortication procedure, the sclerosing OM could be healed uneventfully with bone turnover, resulting in the decrease of bone density in the sclerotic area (the decrease of GSV) with minimal difference from the surrounding healthy bone tissue.

On the other hands, suppurative maxillary OM shows remarkable radiolucency due to soft tissue swelling filled with extensive inflammatory exudate compared to sclerosing OM. As the suppurative OM became healed after surgical enucleation of inflammatory debris and decompressive drain application, its soft tissue density may be rapidly reduced and affect the GSV of panoramic image by increasing GSV in the negative range compared to those not using drain. However, the present data may indicate surgical treatment of suppurative OM using decompressive drain can rapidly remove inflammatory exudate from soft tissue and bone marrow compared to that not using decompressive drain during postoperative period.

The use of negative pressure in wound therapy is a well-known phenomenon that stimulates angiogenesis, improves blood circulation, promotes cell growth of granulation tissue, and accelerates the wound healing process through its mechanical and biological effects in soft tissue. In cases of OM of the jaw, this method has the major advantage of removing inflammatory exudates, including pathogenic bacteria, from areas of postoperative wound edema. This effectiveness was demonstrated in our previous immunoprecipitation high-performance liquid chromatographic analysis study, where the post-operative exudate obtained from the negative-pressure decompression device in cases of chronic suppurative type OM of the jaw showed an increased inflammatory reaction of innate immunity and slight increase of osteogenesis-related proteins such as osteoprotegerin (OPG) and alkaline phosphatase [21]. Cases of BRONJ demonstrated an elevation of inflammatory signaling and increased expression of angiogenesis-related proteins, i.e., VEGF-A and VEGF-C, and osteogenesis-related proteins, i.e., OPG and osteocalcin [22]. In an ex vivo study by Zhang et al., intermittent negative pressure of − 50 kPa inhibited proliferation of human mesenchymal stem cells (MSCs) and promoted cellular apoptosis. In addition, osteoprotegerin ligand mRNA expression was blocked, and OPG mRNA expression increased, resulting in bone regeneration and inhibition of bone resorption [23]. Bone regeneration was demonstrated in a rabbit skull defect by increasing the expression of VEGF and BMP-2 [24]. Therefore, our current radiologic study is consistent with our previous studies and presents the effectiveness of decompression radiographically.

Before undergoing any surgical procedures, a pre-operative laboratory studies such as complete blood count is required for obtaining the necessary information about the patient’s general condition and ensuring the safety of the surgical procedures. It is well established that WBC and ESR are accurate indicators for inflammation. Elevated ESR results is especially useful in the diagnosis and follow-up in patients with osteomyelitis and could be used to monitor the response to therapy [25].

No statistical difference in Hb and Hct was found pre- and post-operatively, which may indicate the usage of present decompressive drains did not affect de novo vascular hemorrhage compared to OM treatment without drain.

In the analysis of inflammatory markers obtained pre- and post-operatively, suppurative OM showed marked decrease of WBC count by using decompressive drain (79.6%) compared to not using (97.2%), while sclerosing OM showed only a little decrease of WBC count by using compressive drain (87.2%) compared to not using (88%). And ANC count was increased in both of sclerosing (86.7%) and suppurative OMs (85.8%) treated with decompressive drain compared to those treated without drain, 80.3 and 82%, respectively. These results indicate the possibility of a residual infection in sclerosing OM and a trend of secondary infection through open wound persisted by drain during postoperative period both in sclerosing and suppurative OMs.

The pre- and post-operative ESR results in suppurative type OM with drain showed significant reduction (48.83%) compared to that of without drain (91.74%). Similarly, the ESR results were significantly different in the sclerosing OM with drain pre- and post-operatively (41.82%), while the sclerosing OM without drain showed no significant difference (69.3%). The reduction in increased laboratory marker for inflammation such as ESR to the normal range could be considered an indicator of successful treatment.

The limitation of our study is associated with the EDPA method. The weak point of EDPA method include the use of panoramic radiographs, where many artifacts and errors could be encountered while converting 3D bone tissue into a 2D depending on the position, the angle and the timing of the photography. The computed tomography (CT), on the other hand is expresses as Hounsfield Unit (HU) and the radiographic bone density can be readjusted as HU’s through a standardization process. Therefore, the results can be used as an absolute value without errors. More precise analysis using periodic CT scan for radiographic bone density measurement and a periodic laboratory data is recommended.

In conclusion, the results of our study indicate positive outcomes of routine use of decompression for management of OM of the jaw. The OM groups with drain exhibited more enhanced bone density compared to the groups without drainage at the six-month and one-year follow-ups. The drain insertion for decompression is effective for the management of sclerosing and suppurative types OM in the jaw. Decompression using the simple draining tubes such as the silastic drain, penrose drain, Jack-Pratt drain and Hemovac have proven to be easy to use, convenient and economical. The intraorally anchored drain compared to the extraoral drain, has more benefits including no scar formation and more comfort to the patient. Based on our study, it is recommended for oral and maxillofacial surgeons to implement decompression with drain for the management of OM of the jaw.

## Data Availability

The datasets used and/or analyzed during the current study are available from the corresponding author on reasonable request.
